# Challenges of Management of Subaortic Membrane in a Young Adult Patient: A Case Report

**DOI:** 10.7759/cureus.48571

**Published:** 2023-11-09

**Authors:** Talal Asif, Lucas A Georger, Krish Sardesai, Maya Kosinska, Muhammad Shah Miran

**Affiliations:** 1 Cardiology, University Health Truman Medical Center, Kansas City, USA; 2 Internal Medicine, University of Missouri Kansas City School of Medicine, Kansas City, USA; 3 Internal Medicine and Pediatrics, University of Missouri Kansas City School of Medicine, Kansas City, USA

**Keywords:** heart failure, guidelines, left ventricular outflow obstruction, aortic stenosis, aortic regurgitation, literature review, case report, management, subaortic membrane

## Abstract

This article presents a case review and literature review focused on the challenges of managing subaortic membranes (SAMs) in young adult patients with mild aortic regurgitation (AR) or aortic stenosis (AS). The study aims to discuss the diagnosis of SAM, the imaging studies used for assessment, the management strategies in young patients, the risk of valvular damage, and the controversy surrounding prophylactic resection in mild AR. The management of SAM in adults poses challenges due to limited treatment options and potential complications, necessitating further investigation into the progression of AS and AR in asymptomatic SAM patients. The case presentation describes a 40-year-old male with muscular dystrophy who presented with symptoms and was diagnosed with SAM. Various imaging techniques, including CT chest, transthoracic echocardiogram (TTE), and transesophageal echocardiogram (TEE), were used to confirm the presence and severity of SAM. Based on the patient's clinical profile and the absence of surgical indications, medical therapy was initiated, and regular outpatient follow-up was recommended to monitor disease progression. The discussion highlights the challenges in diagnosing SAM, the importance of imaging studies, and the potential complications associated with SAM in young patients. The article also explores the management options for SAM, emphasizing surgical resection as the definitive treatment, while acknowledging the limited success rates of alternative approaches. Close monitoring and prompt intervention for complications are crucial in the management of SAM. The concluding statement emphasizes the need for further research to explore alternative treatments for SAM in young patients.

## Introduction

Subaortic membrane (SAM) occurs in the left ventricular outflow tract (LVOT) just below the aortic valve and is characterized by an abnormal fibrous tissue, causing obstruction of blood flow from the left ventricle to the aorta [1]. SAM can cause a range of symptoms, including dyspnea, chest pain, palpitations, and heart failure [1]. Guidelines are clear on the management of SAM if the patient has myocardial ischemia, heart failure, or has a resting peak gradient of >50 mmHg when asymptomatic [2]. However, the guidelines are less clear on the management of adults who present incidentally, have minimal or no symptoms, and have mild aortic stenosis (AS) or aortic regurgitation (AR) that does not meet the threshold of intervention. In addition, there is insufficient guidance on how to pursue surveillance in these patients and the risk of progression of AS and AR is not well known [3]. We present a case and review of the literature to explore the challenges of managing SAM in an adult.

## Case presentation

A 40-year-old Caucasian male with a past medical history of muscular dystrophy initially presented to the emergency department via ambulance after developing abrupt global weakness and dizziness which he said felt was similar to a prior episode of shock following a traumatic spleen rupture. Prior to this episode, he had drunk “a couple of shots” of alcohol but denied being intoxicated. In the emergency department, the patient was bradycardic. EKG showed sinus rhythm; however, the patient was symptomatic. He denied any drug use or blood in the stool. A systolic murmur was noted on a physical exam, best heard at the right second intercostal space. CT angiogram and chest X-ray were negative for aortic dissection, aneurysmal dilation, and pulmonary embolism. The patient was later admitted for symptomatic bradycardia with heart rate in the 40s, mean arterial pressures (MAPs) in the mid-60s, and a low blood pressure of 96/52 in the setting of severe abdominal pain. 

**Figure 1:**
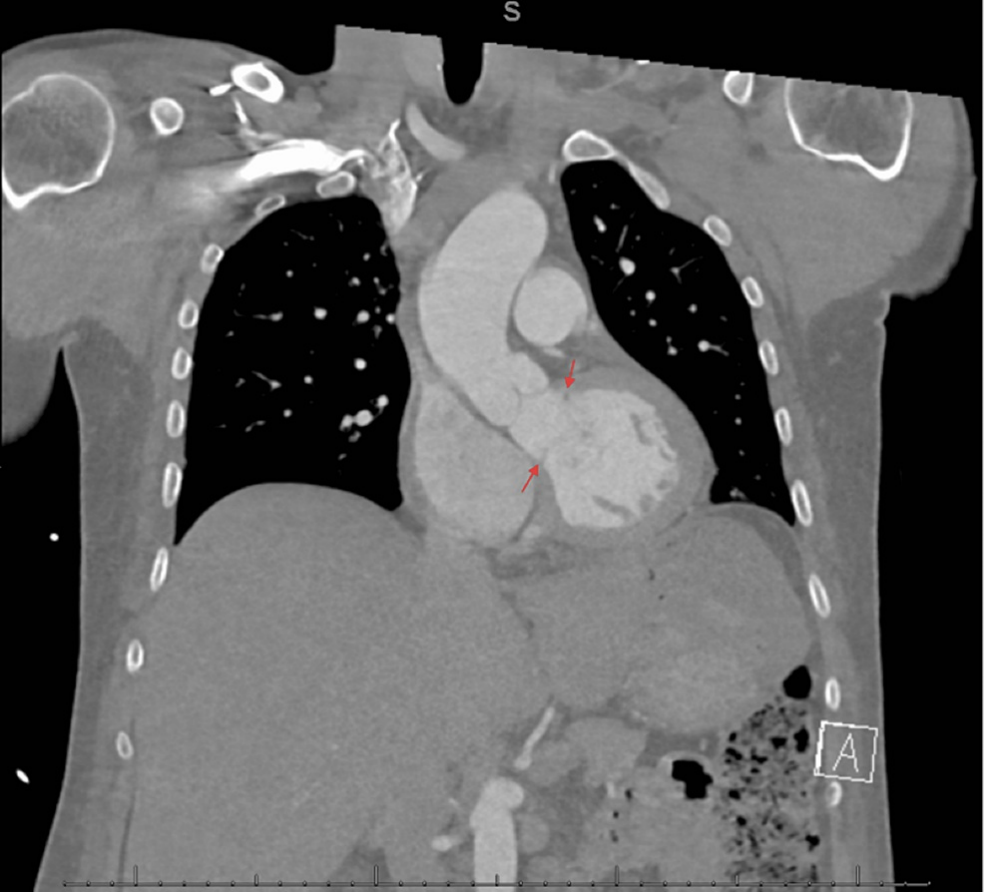
Patient’s CT Chest demonstrating LVOT obstruction. LVOT: left ventricular outflow tract

During his hospital stay, he received IV fluids which resolved the patient’s symptomatic bradycardia. CT chest (Figure 1) was obtained which showed abnormalities in the LVOT. 

**Figure 2:**
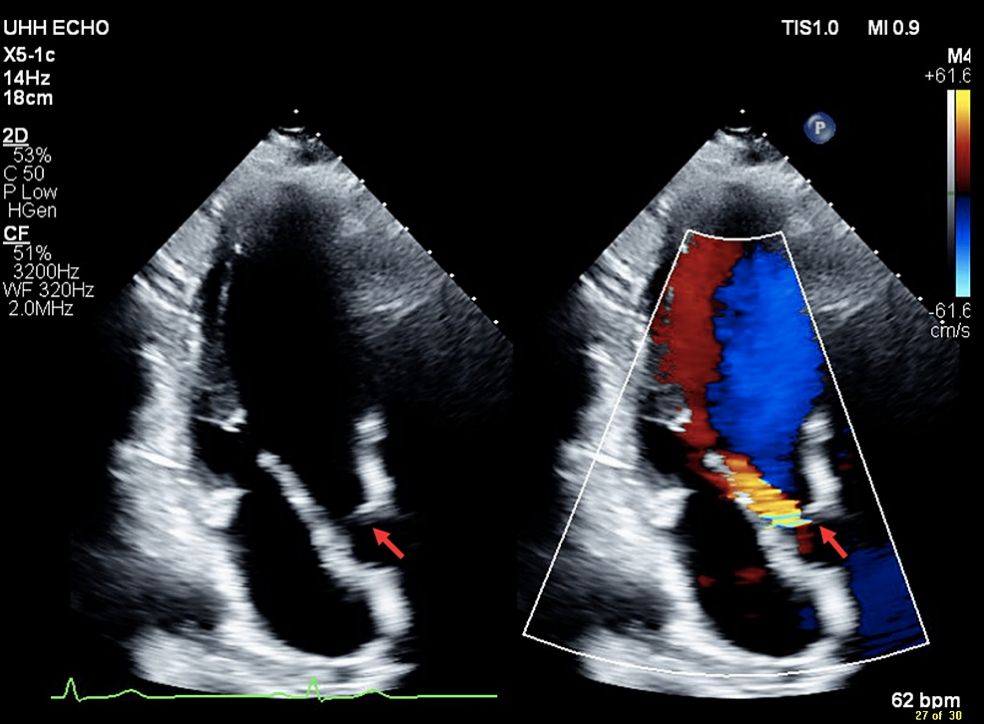
Transthoracic echocardiogram with and without color Doppler demonstrating the presence of a subaortic membrane causing regurgitant blood flow through the aortic valve.

Cardiology was consulted and recommended a transthoracic echocardiogram (TTE) which demonstrated a significant increase in LVOT velocities, with a peak pressure of 31 mmHg and a mean of 18 mmHg. Additionally, the pulmonary artery systolic pressures were between 35 and 40 mmHg. These findings were suggestive of a SAM and mild pulmonary hypertension (Figure 2).

**Figure 3:**
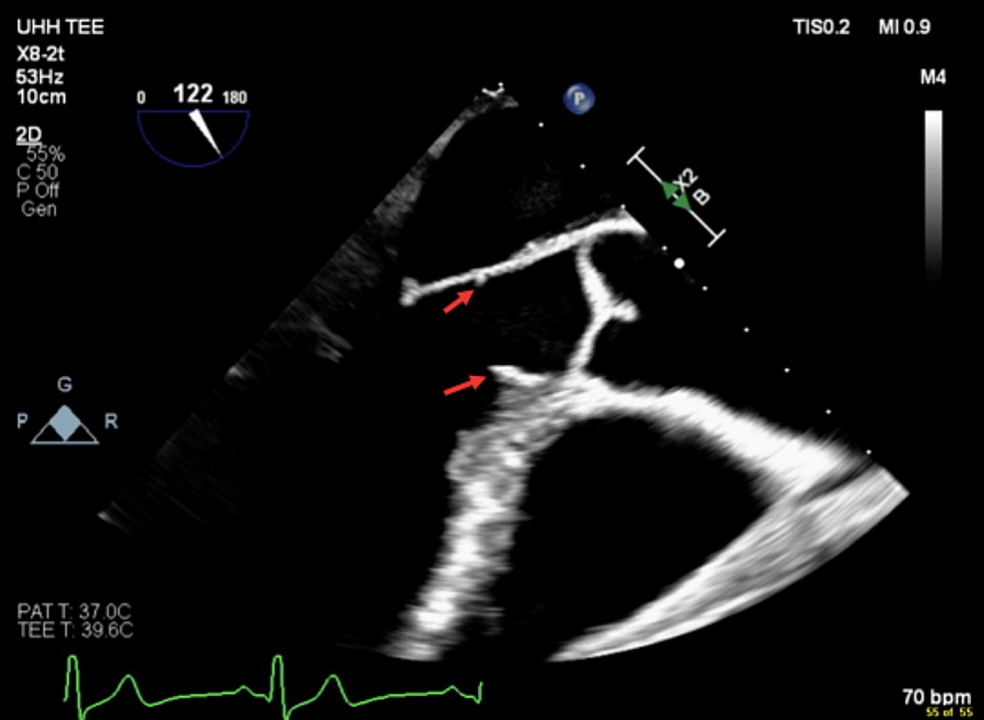
Transesophageal echocardiogram demonstrating the presence of a subaortic membrane.

**Figure 4:**
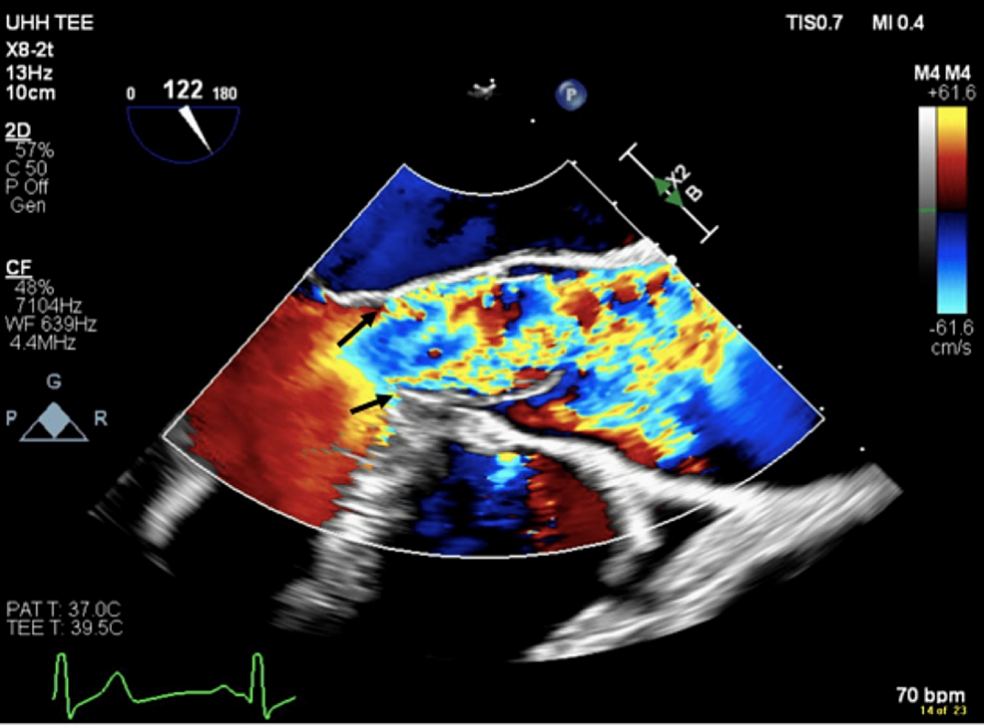
Transesophageal echocardiogram with and without color Doppler demonstrating the presence of a subaortic membrane causing regurgitant blood flow through the aortic valve.

The next day, a transesophageal echocardiogram (TEE) was completed and confirmed the presence of a discrete SAM with peak flows of 22 mmHg across the membrane and mild aortic regurgitation (Figures 3, 4).

The next step in the workup was an exercise stress echocardiogram in order to assess the aortic valve gradients and the patient’s exercise capacity; however, due to the patient’s history of muscular dystrophy, this was not a feasible option. Thus, the cardiology team recommended no further acute intervention as he also did not meet the criteria for surgical correction. At discharge, the patient was medically improved and planned to follow up outpatient with the cardiology clinic to monitor disease progression, which, left unmonitored, may lead to left ventricular hypertrophy, arrhythmias, aortic valve regurgitation, and possible endocarditis [1]. While it is critical these patients are monitored, it is reported that surgical removal of this membrane can reoccur in up to 55% of patients [4]. Reoperation risk factors include female gender, age older than 30 at the time of diagnosis, preoperative peak LVOT gradient greater than or equal to 80 mmHg, and peak LVOT gradient progression over time [1]. This case may help determine the best course of action when treating similar patients, highlighting the importance of diagnostic workup as well as cardiac monitoring post-discharge to minimize risks of disease progression.

## Discussion

Subaortic stenosis secondary to a SAM is a rare structural abnormality frequently found incidentally in cardiovascular workups. Subaortic membranes exist in one of three forms: a thin discrete ridge (most commonly), a fibromuscular ridge, or a diffuse subaortic fibromuscular tunnel-like narrowing of the LVOT, the latter being the most likely form in our patient, although final confirmation requires surgical resection and histopathological analysis [1]. The etiology for SAM is likely genetic and in adults, the course is gradual, as the SAM’s stenosis increasingly obstructs the ejection of blood through the LVOT. In pediatric patients, SAM may progress at a faster and unpredictable rate [1]. Over time, the heart develops concentric hypertrophy with or without a septal bulge, and as the obstruction becomes more clinically significant and severe, patients go from being asymptomatic to developing symptoms such as angina, palpitations, dyspnea, fatigue, syncope, and heart failure [1]. These symptoms vary based on the degree of obstruction caused by the membrane and may worsen during physical activity.

The diagnosis of a SAM can be challenging, as it can mimic other causes of AS or AR, such as hypertrophic cardiomyopathy, bicuspid aortic valve, or rheumatic heart disease [1]. TTE and TEE are vital in identifying the membrane’s anatomy, the severity of LVOT obstruction, and structural damage to the aortic valve [5]. The results of the TTE and TEE are important when considering workup and treatment. Additionally, stress testing for adults with LVOT obstruction to determine exercise capacity, symptoms, electrocardiographic changes, or arrhythmias may be reasonable in the presence of otherwise equivocal indications for intervention [5].

SAM management in pediatric patients is challenging due to limited treatment options and potential complications. SAM can cause valvular damage over time if untreated due to increased turbulent blood flow in the LVOT, causing barotrauma to the aortic valve leaflets and leading to worsening AR [5]. Indications for surgical intervention in pediatric patients are as follows: patients with a Doppler mean gradient <30 mmHg and no left ventricular hypertrophy are not indicated for surgery and should pursue medical follow-up; patients with a Dopper mean gradient >50 mmHg should be surgically treated; and patients with Doppler mean gradient 30-50 mmHg may be indicated for surgery if symptomatic with angina, syncope, or exertional dyspnea or if asymptomatic with ECG changes at rest [1]. There is little consensus regarding the optimal surgical management in pediatric patients, as some advocate solely for membrane resection while others argue for septal myectomy in addition to resection, although recent literature indicates neither strategy shows empirical improvement in reoperation rates [1,6]. In the case of infants, for whom open-heart surgery is acutely contraindicated, they may undergo percutaneous balloon dilatation to provide temporary reduction in aortic gradients and symptoms. This relief may last for a few months to even years, until the patient is more stable, when the open-heart surgery may be performed [1].

Contrary to pediatric patients, in adults, mild AS or AR either progresses slowly or does not progress at all, especially if there is an absence of other congenital abnormalities [3]. This is likely because patients who survive into adulthood are on the lower spectrum of disease severity. 

The 2018 American Heart Association (AHA) guidelines for adults with heart disease state that asymptomatic patients with a peak gradient of 50mmHg or more, or patients who are symptomatic with a max gradient of 30-50mmHg, should be surgically treated [2]. Our patient did not meet this criteria as his TTE revealed gradients of 22 mmHg (peak) and 12 mmHg (mean). Another possible criterion for surgical intervention is if a patient shows signs of cardiac ischemia on exertion [2]; however, this was not possible to evaluate in our patient due to his history of muscular dystrophy. Of note, current literature has not elucidated any clinical connection between limb-girdle muscular dystrophy, seen in our patient, and subaortic stenosis secondary to SAM. In fact, a recent article noted cardiovascular abnormality to be essentially nonexistent in limb-girdle muscular dystrophy [7], further highlighting the uniqueness of our case. Beyond limb girdle, other muscular dystrophies may be associated with cardiac disease, although these abnormalities are more functional than structural, such as conduction defect with arrhythmia [8]. A dobutamine stress test was also considered and scheduled for this patient; albeit, the patient did not make their appointment.

If surgical intervention is indicated, SAM resection is the treatment of choice for AR due to SAM [2]. The timing of intervention is clear in patients who have moderate to severe AR, which constitutes a small subset of these patients. However, prophylactic resection in patients with mild AR is controversial with very limited data suggesting continued observation till the AR becomes hemodynamically significant. The surgery involves excising the SAM and valve replacement or repair [2]. The aortic root can be preserved if it is of normal size, as in this case [2]. Valve replacement is required if the native valve is too damaged to be repaired [2]. This can be accomplished through either transcatheter aortic valve implantation (TAVI) or via surgical aortic valve replacement (SAVR). TAVI is indicated in patients >80 years of age or who pose a great surgical risk, whereas SAVR is preferred in patients <65 years of age or with >20 years of additional life expectancy [9]. When considering immediate workup, surgery should not be delayed in patients with worsening hypotension, pulmonary edema, or low mean arterial pressure [9]. Similar to pediatric patients, additional septal myectomy does not show any enhancement in postoperative outcomes, and due to its increased propensity for complete heart block, is actually contraindicated in adult patients [10]. After aortic valve resection, LV systolic function is an important metric to determine the long-term survival and vitality of the replaced valve [9]. Close follow-up is necessary to monitor for the recurrence of AR.

Few non-surgical interventions exist for SAM as preventative or therapeutic strategies. For SAM patients with a history of endocarditis or previous repair that required a prosthetic device, antibiotic prophylaxis is indicated to prevent bacterial endocarditis. Antibiotic prophylaxis is generally indicated for six months following surgical repair, although it may be indicated for longer in patients with residual defects [1]. Beyond the medical management of SAM, studies are ongoing to evaluate the efficacy of other alternative diagnostic and therapeutic strategies, including minimally invasive heart surgery and genetic testing [11,12].

## Conclusions

Managing SAM in young patients is challenging due to the limited treatment options and potential complications. While imaging and non-surgical workup allow us to make definitive conclusions on the presence and extent of disease, surgical resection remains the definitive treatment. Alternative treatments, such as medical therapy or prophylaxis, minimally invasive heart surgery, and/or genetic testing, have been explored but are currently far from becoming first-line therapy. It is important to closely monitor patients with SAM and promptly address any complications that may arise. Further research is needed to explore alternative treatments for SAM in young patients.
